# Reproducibility Challenges in Fetal Cardiac Function Analysis with 2D Speckle-Tracking Echocardiography: Insights from FetalHQ

**DOI:** 10.3390/jcm14103301

**Published:** 2025-05-09

**Authors:** Jakub Mlodawski, Anna Zmelonek-Znamirowska, Lukasz Pawlik, Marta Mlodawska, Grzegorz Swiercz

**Affiliations:** 1 Collegium Medicum, Jan Kochanowski University in Kielce, 25-516 Kielce, Poland; rjawka@poczta.fm (A.Z.-Z.); marta.mlodawska@ujk.edu.pl (M.M.); grzegorz.swiercz@ujk.edu.pl (G.S.); 2 Clinic of Obstetrics and Gynecology, Provincial Combined Hospital in Kielce, 25-736 Kielce, Poland; 3 Department of Information Systems, Kielce University of Technology, 25-314 Kielce, Poland; lpawlik@tu.kielce.pl

**Keywords:** fetal echocardiography, two-dimensional speckle-tracking (2D STE), FetalHQ, reproducibility, intraclass correlation coefficient (ICC), global sphericity index (GSI), global longitudinal strain (GLS), left ventricle (LV), four-chamber view (4CV), perinatology

## Abstract

**Background/Objectives**: Functional assessment of the fetal heart remains a significant challenge in contemporary perinatology due to the absence of a universally accepted gold standard for such evaluations. The aim of this study was to evaluate the reproducibility of parameters derived from two-dimensional speckle-tracking echocardiography (2D STE) using the FetalHQ software. **Methods**: We enrolled 87 pregnant women between 19 and 23 weeks of gestation who were undergoing mid-trimester screening at the Provincial Hospital Complex in Kielce. Two independent operators acquired 5 s cine-loops of four-chamber views (4CVs) according to a standardized protocol. Reproducibility was assessed by examining intra- and interobserver variability using the intraclass correlation coefficient (ICC) for several cardiac parameters, including the global sphericity index (GSI), global longitudinal strain (GLS), stroke volume (SV), and fractional area change (FAC). **Results**: Reproducibility varied substantially across the assessed parameters. The highest intraobserver reproducibility was observed for the 4CV GSI (ICC > 0.9). Moderate intraobserver reproducibility (ICCs ranging from 0.5 to 0.75) was noted for left ventricular (LV) parameters, such as end-diastolic area, end-systolic area, end-diastolic volume, and end-systolic volume. Interobserver variability demonstrated higher ICC values, with excellent reproducibility (ICC > 0.9) for the 4CV GSI and LV volume measurements. Good reproducibility (ICCs between 0.75 and 0.9) was observed for specific left ventricular segmental strain indices, whereas other parameters showed moderate to poor reproducibility (ICC < 0.5). **Conclusions**: Two-dimensional speckle-tracking echocardiography (2D STE) using FetalHQ exhibits variable reproducibility, which is influenced by the choice of parameters, operator experience, and technical factors. This method holds potential for fetal cardiac assessment; however, additional research is required to improve its predictive accuracy and streamline the evaluation process for routine clinical application.

## 1. Introduction

In fetal screening and specialized ultrasound examinations, considerable emphasis is placed on evaluating cardiac structure due to the high prevalence of congenital heart defects in the general population. However, certain maternal complications (e.g., gestational diabetes) and fetal conditions (e.g., fetal growth restriction) can induce functional cardiac alterations in the absence of structural abnormalities [1,2,3].

Traditionally, cardiac function in adult echocardiography has been assessed using left ventricular ejection fraction (LVEF). This approach, however, is limited by assumptions such as a symmetrical left ventricular shape and a smooth endocardial surface. Furthermore, LVEF is influenced by heart rate, highlighting the need for more refined methods to evaluate ventricular function [4]. Due to the distinct nature of fetal circulation, intrauterine diagnostics also necessitate the assessment of the right ventricle [5].

Commonly used Doppler-based methods for evaluating fetal cardiac function include the myocardial performance index (MPI, or Tei index) and the E/A ratio. These parameters have demonstrated predictive capabilities for neonatal outcomes in complicated pregnancies, such as gestational diabetes or FGR [6,7]. However, their angle-dependent nature and susceptibility to fetal blood rheology variations limit their reliability [1].

Myocardial strain (MS) has emerged as a metric for evaluating cardiac function, reflecting myocardial deformation as a dimensionless unit expressed as a percentage (i.e., the change in fiber length during the cardiac cycle). MS measurement relies on detecting frequency shifts in waves reflected from moving objects. Speckle-tracking echocardiography (STE) tracks natural myocardial reflections (“speckles”) across frames, providing measures of strain—indicating muscle fiber deformation—and the strain rate, which reflects the rate of this deformation [4]. STE enables the assessment of longitudinal, circumferential, and radial strain in myocardial fibers and can be applied to both two-dimensional (2D) and three-dimensional images. Unlike Doppler-based methods, STE is less dependent on angle, facilitating measurements in multiple directions. However, its sensitivity to image quality poses a challenge, particularly in fetuses of mothers with obesity. Additional limitations include higher proximal speckle-tracking values compared to distal ones and the restriction of 2D STE to single-plane measurements at a time [4]. This technique also supports offline analysis following prior scan acquisition. STE also supports offline analysis, and because tracked points are located on the endocardial surface—highly sensitive to hypoxia—it may offer predictive benefits in assessing outcomes and monitoring fetuses with conditions such as FGR or vascular abnormalities. The primary variable in these assessments is strain, defined as the percentage displacement of the examined acoustic marker. While strain analysis is well established in adult echocardiography, its application in fetal echocardiography remains limited. Recent recommendations from the American Society of Echocardiography list strain assessment among Alternative Ultrasound Modalities, noting that its clinical use during pregnancy requires further investigation [5].

Studies on 2D STE have reported differences in parameters among fetuses in pregnancies complicated by FGR, including abnormal longitudinal strain, strain rate, and dyssynchrony [8]. Additionally, abnormal 24-segment sphericity indices for both ventricles have been observed in small-for-gestational-age fetuses, highlighting the potential of STE for detecting subtle functional changes [9]. In pregnancies complicated by gestational diabetes, evidence suggests systolic dysfunction, particularly in the right ventricle [10]. However, published studies exhibit significant heterogeneity in design, complicating direct comparisons and definitive conclusions. The variety of systems used for fetal 2D STE, such as Velocity Vector Imaging (VVI, Siemens), STE (TomTec), STE EchoPAC (GE), and WMT TestDriver (Toshiba), further limits the comparability of results. No studies have directly compared these systems for reproducibility in the same patient [8,10].

The Fetal Heart Quantification (FetalHQ) software is a semi-automated tool designed for speckle-tracking analysis in four-chamber views (4CVs) of the fetal heart. It evaluates a wide range of parameters and supports multi-segment analysis, dividing both ventricles into 24 segments from their basal to apical regions. During FetalHQ analysis, the algorithm first guides the user through the assessment of the global sphericity index (GSI), a parameter derived solely from the static morphology of the heart in a 4CV (calculated as End-Diastolic Length/End-Diastolic Width). FetalHQ is a sophisticated tool but, in our opinion, is prone to operator-dependent measurement errors at various stages. Assessing the reproducibility of this technique is essential prior to its clinical implementation. There remain numerous unexplored opportunities in perinatology where such a functional assessment could be applied—from diagnosing fetal cardiac dysfunction and monitoring fetuses in complicated pregnancies (e.g., fetal growth restriction, hypertension, and diabetes) to evaluating fetuses after invasive procedures and supporting research studies.

## 2. Aim of This Study

The objective of this study was to assess the reproducibility of parameters derived from two-dimensional speckle-tracking echocardiography (2D STE) using the FetalHQ software.

## 3. Materials and Methods

This study included patients undergoing routine mid-trimester screenings at the Gynecology and Obstetrics Clinic of the Provincial Hospital Complex in Kielce. The study was approved by the Bioethics Committee at Jan Kochanowski University in Kielce (Approval No. 35/2024, approval date: 8 May 2024). All procedures adhered to relevant local regulations and guidelines established by the ethics committee. Informed consent for ultrasonographic evaluation and study participation was obtained from all participants. Examinations were performed using a Voluson Expert 22 ultrasound system equipped with a Convex Probe C2-9-D and the latest version of the FetalHQ software (September 2024).

The study enrolled patients with singleton pregnancies at gestational ages typical for mid-trimester screening (19–23 weeks). Participants were consecutively recruited. Exclusion criteria included fetuses with abnormal heart imaging during screening, multiple pregnancies, and patients who declined participation. In addition to FetalHQ data, we collected information on maternal age, gestational age, weight, height, and body mass index (BMI).

Two operators (J.M. and A.Z.-Z.) independently evaluated fetal cardiac contractility using speckle-tracking technology, following the manufacturer’s guidelines for FetalHQ [11]. The operators performing the examinations had over seven years of experience in prenatal diagnostics. Each operator acquired a cine-loop of the four-chamber view (4CV), standardized according to the International Society of Ultrasound in Obstetrics and Gynecology (ISUOG) guidelines for fetal heart screening, with adjustments for FetalHQ image acquisition specifications [12]. The image was magnified to occupy the full screen in the 4CV, with the beam angle minimized to achieve a high frame rate (utilizing the second-trimester fetal heart preset). To reduce dropout effects on the interventricular septum, the heart was oriented with the apex nearly perpendicular to the ultrasound beam (encompassing perpendicular and oblique axes). Any 4CV images permitting only apex-up or apex-down orientations were excluded, and patients with such orientations were not enrolled. Upon freezing the image in the 4CV scan, the FetalHQ software guided the operator through the assessment process, which involved the following steps:

(1). The operator measured two perpendicular cardiac dimensions—length (4CV Length, from the apex to the base of the cardiac outer edge) and width (4CV Width, from the sidewall of the left ventricle [LV] to the sidewall of the right ventricle [RV] at end-diastole)—to calculate the global sphericity index (GSI).

(2). Subsequently, the operator traced an M-mode line from the apex along the lateral wall of the right ventricle, passing through the tricuspid valve leaflet, ensuring no image drift in M-mode. Within a single cardiac cycle, three points were marked: (1) end-diastole at the start of the cardiac cycle, (2) end-diastole at the end of the cycle, and (3) end-systole, corresponding to the maximum closure of the atrioventricular valves. Following recommendations, end-diastolic tracing of the endocardial borders of both the left and right ventricles was then performed.

(3). The tracing process began by placing three red points: (1) the septal attachment of the valve, (2) the valve attachment on the free wall, and (3) the endocardium at the cardiac apex. Per the guidelines, the papillary muscle and moderator band were included in the outline. The software automatically traced the endocardial borders of both ventricles in end-systole, starting from the valve attachment points and extending to the apical point, with manual adjustments available for inaccurate outlines. The ventricular area was then divided into 24 segments parallel to the heart’s base, followed by image analysis.

(4). In each case, the software evaluated contractility across 24 segments of both the right and left ventricles, generating parameters such as global longitudinal strain (GLS), fractional area change (FAC), 24-segment end-diastolic diameter (EDD), fractional shortening (FS), and sphericity index (SI) for both ventricles. These parameters were automatically adjusted to centile charts by the software. Notably, fetal biometric measurements were conducted only once, even when examinations involved two operators. Results of the segmental analysis, calculated for 24 segments of each cardiac chamber in FetalHQ, are presented in this study for only three segments—basal (segment 1), mid-ventricular (segment 12), and apical (segment 24)—due to the volumetric constraints of this publication. This selection is driven by two factors: first, the necessity of arbitrarily choosing data for presentation in this manuscript, and second, the specific characteristics of individual segments, which may influence the reproducibility of the analysis results. The apical segment includes the papillary muscle, which should be contoured at its base, while part of it extends into the lumen of this segment. Conversely, the basal segment encompasses the valve attachments within its contour, with the valve leaflets positioned within the segment’s lumen. These factors may affect the quality of the contours drawn prior to analysis, as irregularities in the contour and potential blurring of boundaries for mobile structures could occur. The mid-ventricular segment was selected because it appears to be the most straightforward to contour. Additionally, segments were chosen to be as spatially distant from each other as possible, based on literature evidence suggesting that the physiology of individual segments may differ in cases of certain pregnancy complications [13]. Reliability results for the remaining segments are provided in the Supplementary Materials of this study and are deposited in a public repository (https://doi.org/10.17605/OSF.IO/SFA7T (accessed on 26 March 2025)).

For intraobserver variability assessments (by a single operator), each operator independently analyzed the cine-loop, repeating the described steps twice after the patient session, with an interval of several days between assessments to avoid immediate-repetition bias. For interobserver variability assessments (by two operators), both operators analyzed the same cine-loop, previously acquired by the first operator. The second operator was blinded to the results of the initial measurement. Cine-loops, approximately 5 s in duration, were used. The raters’ repeated measurements were not averaged during the examination, as the study aimed to reflect real-world clinical conditions. Additionally, the cardiac cycle analyzed within the 5 s cine-loop was randomly selected by the operator. While beat-to-beat variation is acknowledged in real-world settings, this phenomenon should not account for differences in measurements within the same fetus if the measurements are reliable.

## 4. Statistical Analysis

For demographic descriptions of the study population, measures of central tendency are reported as either the median or arithmetic mean, depending on the normality of the variable distribution, which was assessed using the Shapiro–Wilk test. Measures of dispersion are expressed as the interquartile range (IQR) or standard deviation (SD), as appropriate. To evaluate reproducibility, the intraclass correlation coefficient (ICC) was calculated using a two-way mixed-effects model, accounting for two sources of variability (operators and subjects [cine-loop clips]), consistent with the established literature [14]. Previous studies reporting ICC values for parameters derived from Fetal Heart Quantification (FetalHQ) have indicated a minimum ICC of 0.4 [14,15]. Accordingly, based on sample size tables provided by Bujang et al. [16], with a null hypothesis ICC of 0.5, a minimum alternative hypothesis ICC of 0.7, an alpha level of 0.05, and a statistical power of 0.9, a minimum sample size of 30 patients per study arm was determined. Initially, 123 patients were enrolled, but 29% were excluded due to apex-up or apex-down heart orientations. Ultimately, 87 patients were included (34 for intraobserver variability and 53 for interobserver variability).

Each ultrasound (USG) examination generated 1420 individual parameters. The volume and complexity of these measurements posed challenges for clinical interpretation, necessitating computational solutions to automate data processing as much as possible. The USG software exported results as CSV files, with rows representing either group headers or individual measurement outcomes. Advanced programming techniques were employed for data analysis. Statistical analysis was conducted using SPSS 27.0.1.0 (IBM Corporation) alongside three custom Python 3.12.2 programs, leveraging the Pandas, NumPy [17], and Pingouin [18] libraries:

**IMPORT-EXAM Program**: This program independently imported USG results from each dataset and exported two CSV files per dataset, each containing rows corresponding to individual examinations for a single patient.

**MERGE-EXAM Program**: This program consolidated examination data from each dataset into a single CSV file. For each patient, the file included paired measurement results from the examinations.

**CALC-ICC2 Program**: This program read the merged CSV file for each dataset, computed ICC values, and exported the results as CSV files.

The CALC-ICC2 program processes only numerical data, transforming them into a “long” format where each measurement value occupies a separate row, with columns identifying the patient and measurement type. The program iterated through each detected parameter (e.g., LV Global Strain, RV Global Strain). For each parameter, it verified that the number of non-missing values was at least five; parameters with fewer non-missing values were excluded. Additionally, the program assessed data variance, excluding measurements with zero variance (i.e., identical values across observations); however, this scenario did not occur in our dataset. ICC calculations proceeded only when these criteria were met. The ICC was computed using the “intraclass_corr” function, ensuring consistent data processing and minimizing errors, thereby enhancing the precision and reliability of the study results. The generation of intermediate files further enabled the analysis of both final and intermediate outcomes. ICC values were interpreted as follows: values below 0.5 indicated poor reliability, 0.5 to 0.75 indicated moderate reliability, 0.75 to 0.9 indicated good reliability, and values above 0.9 indicated excellent reliability [12]. A *p*-value < 0.05 was considered statistically significant.

Absolute observer agreement (inter- and intrarater agreement) was also assessed for specific measurements based on whether the results fell within the 5th to 95th percentile range or a Z-score range of −1.645 to 1.645 (expressed as overall percentage agreement). Percentile values were obtained from the FetalHQ software. Agreement levels were classified as follows: above 90% was deemed excellent, 70–90% was considered good, and below 70% was regarded as poor.

## 5. Results

The study enrolled 87 patients undergoing mid-trimester screening at the Gynecology and Obstetrics Clinic between 19 and 23 weeks of gestation (34 patients for intraobserver variability assessment and 53 for interobserver variability assessment). Given the extensive data generated by a single FetalHQ analysis (up to 1420 variables), only a subset of results is presented in this article. For parameters involving 24 segments, data are reported for the basal segment (segment 1), the apical segment (segment 24), and an intermediate segment (segment 12). These segments were selected to represent the two extremes and a midpoint. The complete dataset, normalized against centile charts for each anthropometric parameter, is available along with dataset (https://doi.org/10.17605/OSF.IO/SFA7T (accessed on 26 March 2025)). 

To meet technical requirements, 123 patients were initially screened to achieve the final study cohort; 29% of the initial group was excluded due to apex-up or apex-down heart orientations. In these fetuses, an appropriate projection could not be obtained, which, per the manufacturer’s instructions, precluded accurate analysis [11].

Demographic characteristics of the pregnant women are presented in Table 1. Selected results from the reproducibility analysis are shown in Table 2. Additional data are included in the statistical analysis results, which, along with the full dataset, have been assigned the https://doi.org/10.17605/OSF.IO/SFA7T (accessed on 26 March 2025).

The results revealed considerable variability in reproducibility depending on the measured parameter. The highest intraobserver reproducibility was observed for the global sphericity index (GSI) measurement. Moderate intraobserver reproducibility was noted for volumetric parameters of the left ventricle, including left ventricular end-diastolic area (LV ED Area), left ventricular end-systolic area (LV ES Area), left ventricular end-diastolic volume (LV EDV), and left ventricular end-systolic volume (LV ESV). Intraclass correlation coefficient (ICC) values for other parameters indicated poor intraobserver reliability.

For interobserver variability, higher ICC values were observed. Excellent reproducibility was achieved for the four-chamber view global sphericity index (4CV GSI), LV ED Area, LV ES Area, LV EDV, and LV ESV. Good ICC values were recorded for the left ventricular sphericity index (SI) in segments 1 and 12. Moderate reliability was observed for left ventricular stroke volume (LV SV), left ventricular cardiac output (LV CO), left ventricular global longitudinal strain (LV GLS), and SI in segment 24, while the remaining parameters exhibited poor reliability. Table 3 presents the overall percentage agreement between measurements conducted by two different operators and repeated measurements by the same operator. A good overall percentage agreement was achieved for the majority of the parameters evaluated.

## 6. Discussion

Assessing fetal cardiac function is critical for several reasons. Functional changes in the heart may represent early manifestations of cardiac or systemic fetal pathologies, potentially progressing to circulatory system dysfunction. Such evaluations can also facilitate the monitoring of disease progression. Conditions where this assessment is particularly valuable include structural congenital heart defects, fetal anemia, and multiple pregnancies complicated by twin-to-twin transfusion syndrome (TTTS), among others [19]. Challenges in accurately assessing cardiac function arise from the heart’s complex biomechanics and the orientation of muscle fibers across multiple planes, resulting in shearing, shortening, rotation, twisting, and torsion [20]. Furthermore, the heart’s irregular shape, along with structures such as papillary muscles, chordae tendineae, and valve leaflets protruding into the ventricular lumen, complicates precise functional evaluation [20].

Evaluating fetal cardiac contractility during pregnancy remains a significant challenge in modern perinatal medicine, primarily due to the absence of a gold-standard diagnostic test. Historically, M-mode echocardiography was employed to measure systolic-diastolic differences in the ventricles, enabling the calculation of fractional shortening (FS) [21]. M-mode also allows the assessment of tricuspid annular plane systolic excursion (TAPSE) and mitral annular plane systolic excursion (MAPSE) by measuring the displacement of the tricuspid and mitral valve leaflets. However, due to the assumptions of the method, these assessments will always be one-dimensional and angle-dependent.

Another approach involves using color Doppler imaging to assess valvular blood flow and measure valve areas, facilitating the calculation of stroke volume (SV) and, with heart rate, cardiac output [21]. Doppler techniques also enable the evaluation of the E/A ratio, an indicator of fetal diastolic function. Additionally, the Tei index (myocardial performance index, MPI), derived from isovolumetric contraction and relaxation times and ejection time, provides a comprehensive measure of both systolic and diastolic function [3]. A key advantage of the Tei index is its dual assessment capability; however, all Doppler-based methods are angle-dependent, and fetal conditions affecting blood rheology can influence their outcomes [1]. Consequently, speckle-tracking analysis has garnered significant interest as a promising alternative.

For novice FetalHQ users, despite step-by-step instructions, the volume of data generated can be overwhelming. Most parameters are calculated across 24 segments for both the right and left ventricles, with some normalized to centile charts based on fetal biometric measurements (e.g., head circumference, biparietal diameter, abdominal circumference, femur length, estimated fetal weight, and gestational age). Thus, the software simultaneously assesses chamber size, shape, and contractility. However, even with guided steps, this examination poses challenges for new users. Operators require experience in fetal heart screening to obtain an optimal dynamic four-chamber view (4CV) scan capturing multiple cardiac cycles without fetal movement or apex-up/down positioning, which can cause dropout effects. Proficiency in ultrasound probe manipulation is also essential to align the interventricular septum as perpendicular to the ultrasound beam as possible. In practice, nearly one-third of fetuses require extended examination, maternal repositioning, or deferred assessment due to suboptimal fetal positioning. The recorded cine-loop, containing several cardiac cycles, is critical for intraobserver variability assessment; in this study, offline clips approximately 5 s in duration were analyzed. Variability may arise from differences in M-mode line placement (each assessment involved separate line positioning) and the selection of different cardiac cycles. In our study, evaluations were independent, with the second observer blinded to the cardiac cycle chosen by the first. For intraobserver variability (repeated assessments by the same ultrasonographer), consistency in M-mode line placement and cycle selection was more likely, as the observer might recall previous measurements.

We propose that the primary factor contributing to low intraclass correlation coefficient (ICC) values in this study was endocardial tracing. While the FetalHQ system largely automates this process—requiring the operator to place three manual points per ventricle, after which the software outlines the endocardium and delineates 24 segments—adjustments are possible. Operators can reposition the initial red points to adjust the entire tracing line or move individual blue points to modify specific segments, affecting adjacent lines. Variability in endocardial tracing between observers may stem from physical limitations such as attenuation, acoustic shadowing, reverberations, and diffraction, which can obscure the endocardial boundary, particularly in ventricles farther from the probe. The irregular ventricular surface further complicates endocardium delineation, especially near the apex. Per consensus guidelines, papillary muscles and the moderator band should be included within the outlined contour [11], adding to the difficulty of achieving consistent results. These factors, combined with broad numerical ranges, large datasets (including parameters normalized to inherently variable biometric data), and a complex method with a steep learning curve, suggest that this test may not be feasible for all fetuses. In our study, 29% of patients were excluded due to apex-up/down orientations, aligning with literature findings. Semmler et al. [22] reported that 32.9% of fetuses in their study on the impact of heart orientation on global longitudinal strain (GLS) analysis exhibited apex-up/down positioning, indicating that a substantial proportion of fetuses may be ineligible for this assessment or require waiting for optimal fetal positioning.

FetalHQ reproducibility has received limited attention in the literature, with few studies addressing this topic. Gireadă et al. [15] evaluated intra- and interrater reliability, reporting ICC values for end-diastolic (ED) and end-systolic (ES) area and length measurements exceeding 0.75 within raters and 0.5 between raters. Good reliability was observed for left ventricular fractional area change (LV FAC) and GLS, whereas right ventricular (RV) measurements yielded lower values. This discrepancy may reflect several factors. The cine-loop duration in Gireadă et al. [15] was shorter (3 s) than in our study (~5 s), increasing the likelihood of selecting the same cardiac cycle for interobserver variability assessments. Beat-to-beat variation is a known limitation in fetal cardiac function, and FetalHQ parameters may vary across cycles. However, for clinical utility, the method should demonstrate reproducibility regardless of the cycle selected. Repeated measurements with averaging would significantly extend the examination time, which is already considerable. Additionally, a previous study [15] studied larger fetuses in the third trimester, though the impact of gestational age on 2D STE analysis using FetalHQ remains unclear due to limited data. Operator experience may also influence outcomes; in our study, scans were performed by obstetricians with over seven years of experience in second-trimester prenatal screening, rather than fetal echocardiography specialists. The experience level of operators in that study was not specified [15]. Despite detailed manufacturer guidelines, most FetalHQ measurements yielded unsatisfactory ICC values, potentially leading to reporting differences between centers, particularly when establishing cut-off points for pathologies. Low intraclass correlation coefficient (ICC) values may hinder progress toward the full clinical implementation of the method in routine medical practice. This is due to variations observed across sequential examinations conducted over time and between operators, which consequently pose challenges in interpreting whether observed changes in values reflect a trend indicative of fetal deterioration or improvement, or if they result from limitations in the method’s reproducibility. Future research should focus on investigating the relationship between these two variables.

A 2021 study [23] reported excellent ICC values for ventricular area estimation, with lower values for the global sphericity index (SI) (0.78), LV SI (0.65), RV SI (0.58), LV fractional shortening (FS) (0.53), and RV FS (0.36). Although these ICC values exceed those in our study, the pattern of reproducibility—from highest to lowest—remains consistent. A 2022 study in a Japanese population [24] reported poorer reproducibility, with not all ICC values reaching statistical significance. For interobserver variability, SI values for left ventricular segments ranged from 0.473 to 0.755, and for the right ventricle, from 0.48 to 0.767. The researchers noted better reproducibility for RV assessments and observed differences between basal and apical segments, with higher intra- and interobserver variability for apical segments, mirroring our findings. Variations among studies may result from methodological differences, such as the exclusion of apex-up/down heart orientations in our study. We suggest that challenges in RV reproducibility partly stem from its anatomical structure, characterized by a more trabeculated wall and the presence of the moderator band on ultrasound.

Low reproducibility for certain parameters may also arise from their derivation through mathematical equations involving multiple measurable variables, each prone to measurement error. For example, SI is calculated as ED length/ED transverse diameter, and FS as ((EDD − ES diameter) × 100/EDD). Another key factor in segmental analysis is the method’s sensitivity to marker placement. Minor adjustments in marker position can shift segment locations, altering ventricular division. Consequently, SI values for a theoretically identical segment may correspond to different ventricular regions between operators. Similarly, FAC exhibited low reproducibility in our study. As a global ventricular parameter reliant on two measurements—(ED area − ES area) × 100/ED area—assessed at distinct cardiac cycle points, it requires two separate endocardial tracings, compounding measurement error and reducing reproducibility.

Our study achieved slightly better results for absolute agreement between examiners, a notable finding given current trends in biological sciences. Research on predictive models suggests that FetalHQ-derived variables could be binarized (e.g., normal/abnormal) and integrated into such models. However, with the large number of parameters per examination, a larger study population is required, which poses challenges, particularly for predicting rare cardiac defects. A regression-based approach to interpreting results or reducing dimensionality by identifying correlations appears to be well justified. This strategy has been documented in the literature. DeVore et al. [25] developed a multiparameter model based on 4CV data from FetalHQ, achieving 96% sensitivity and an area under the receiver operating characteristic curve (AUC) of 0.98 for diagnosing aortic coarctation (CoA). In contrast, current ultrasound methods (e.g., aortic isthmus/arterial duct diameter ratio) exhibit lower diagnostic sensitivity [26]. Similar models have been described for diagnosing tetralogy of Fallot (ToF), D-transposition of the great arteries (dTGA), and predicting the need for emergency balloon neonatal septostomy in fetuses with restrictive oval foramen and dTGA [27,28,29].

The natural progression of 2D Spatio-Temporal Image Correlation (STIC) FetalHQ appears to be 4D STIC. Preliminary studies evaluating 4D STIC reproducibility have demonstrated good reliability for ventricular morphological parameters, with slightly lower ICC values (<0.8) for SI and FS [30]. In certain areas of medicine, speckle-tracking echocardiography (STE) has already established its clinical utility; however, in the field of maternal–fetal medicine, we are still at the early stages of integrating STE into routine clinical practice. Previous studies have yielded inconsistent results regarding physiological changes in STE parameters over the course of pregnancy (such as longitudinal strain and strain rate). One of the limitations in STE assessment appears to be the wide variety of available systems, coupled with a lack of studies directly comparing these platforms in the same fetal subjects. Technical factors such as frame rate, spatial resolution, and beat-to-beat variation may also influence the analysis [31]. Further research is warranted to identify critical moments during the examination procedure that, in real-world practice, may hinder the achievement of the desired high reliability.

A strength of our study is its strict adherence to the manufacturer’s guidelines for FetalHQ software. This study adhered strictly to FetalHQ guidelines and focused on methodology rather than fetal heart pathology. All fetuses were assessed at similar gestational ages, and 4CV images with apex-up/down orientations were excluded to minimize positional effects on the results. This study also reflects real-world conditions and was conducted by practicing clinicians. However, the limitations include a relatively small sample size, though it aligns with sample size calculations, and the lack of consideration of potential confounders like BMI or subcutaneous tissue thickness. This study was restricted to healthy fetuses without structural heart abnormalities. Additional limitations include its single-center design and the monoethnic study population, which may limit generalizability.

## 7. Conclusions

Two-dimensional speckle-tracking echocardiography (2D STE) using FetalHQ exhibits variable reproducibility depending on the parameter evaluated. Offline reproducibility between operators was found to be inadequate. This limitation may be attributed to the complexity of the study, multiple technical factors affecting outcomes, and the influence of operator experience. Further research is warranted to assess FetalHQ in both healthy and pathological fetal populations and to evaluate the predictive value of specific parameters. Such studies are essential to streamline the examination process and enhance result interpretation for clinical application.

## Figures and Tables

**Table 1:** Demographic characteristics of the study group.

Parameter	Value
Age [mean, SD]	29.24 (±5.45)
Gestational age [median, IQR]	21 (1.2)
BMI [kg/m^2^]	25.3 (3.1)

**Table 2:** The results of the reproducibility analysis, including 95% confidence intervals. The table presents the intraobserver correlation coefficient (ICC) along with the confidence interval (CI) for various parameters: four-chamber view global sphericity index (4CV GSI), left ventricular (LV) and right ventricular (RV) global longitudinal strain (GLS), stroke volume (SV), cardiac output (CO), end-diastolic velocity (EDV), end-systolic velocity (ESV), strain index (SI), and fractional shortening (FS).

	Intraobserver Variability		Interobserver Variability	
Parameter	ICC	95% CI	ICC	95% CI
4CV GSI	0.97	0.93–0.98	0.99	0.99–1.00
LV GLS [%]	0.35	0.01–0.61	0.5	0.27–0.68
RV GLS [%]	0.21	0.776–0.884	0.28	0.02–0.51
LV Fractional Area change [%]	0.08	−0.27–0.4	0.46	0.22–0.65
RV Fractional Area change [%]	0.27	−0.06–0.55	0.23	−0.05–0.47
LV_ SV [mL]	0.2	−0.15–0.51	0.69	0.52–0.81
LV_ CO [mL/min]	0.33	−0.01–0.6	0.53	0.3–0.7
LV_ ED Area [cm]	0.54	0.25–0.74	0.94	0.9–0.96
LV_ ES Area [cm]	0.7	0.41–0.81	0.91	0.85–0.95
LV_ EDV [mL]	0.57	0.3–0.76	0.94	0.89–0.96
LV_ ESV [mL]	0.57	0.29–0.76	0.91	0.86–0.95
LV 24-Segment SI Values [%] Segment 1	0.18	−0.17–0.49	0.89	0.81–0.93
LV 24-Segment SI Values [%] Segment 12	0.49	0.18–0.71	0.88	0.8–0.93
LV 24-Segment SI Values [%] Segment 24	0.37	0.03–0.63	0.61	0.42–0.76
RV 24-Segment SI Values [%] Segment 1	0.36	0.03–0.62	0.26	0.00–0.48
RV 24-Segment SI Values [%] Segment 12	0.48	0.17–0.7	0.45	0.21–0.64
RV 24-Segment SI Values [%] Segment 24	−0.02	−0.36–0.33	0.27	0.01–0.51
LV 24-Segment FS Values [%] Segment 1	0.07	−0.28–0.4	0.33	0.07–0.55
LV 24-Segment FS Values [%] Segment 12	0.26	−0.09–0.55	0.38	0.13–0.59
LV 24-Segment FS Values [%] Segment 24	0.25	−0.1–0.54	0.32	0.05–0.54
RV 24-Segment FS Values [%] Segment 1	0.11	−0.22–0.42	0.16	−0.12–0.41
RV 24-Segment FS Values [%] Segment 12	0.33	0–0.59	0.24	−0.04–0.48
RV 24-Segment FS Values [%] Segment 24	0.44	0.14–0.67	0.28	0.01–0.51

**Table 3:** Inter- and intrarater absolute agreement across individual cases, showing agreement based on qualitative data (i.e., results falling within the 5th–95th percentile range or a Z-Score range from −1.645 to 1.645) depending on the parameter assessed.

	Interrater Agreement	Intrarater Agreement
	Z-Scores (−1.645 to 1.645) agreement	
RV 24-Segment ED Z-Scores (EFW)[] Segment 1	82.35%	79.25%
RV 24-Segment ED Z-Scores (EFW)[] Segment 14	64.71%	79.25%
RV 24-Segment ED Z-Scores (EFW)[] Segment 24	52.94%	81.13%
RV 24-Segment SI Z-Scores[] Segment 1	64.71%	71.7%
RV 24-Segment SI Z-Scores[] Segment 14	73.53%	71.7%
RV 24-Segment SI Z-Scores[] Segment 24	73.53%	84.91%
LV 24-Segment ED Z-Scores (EFW)[] Segment 1	76.47%	71.7%
LV 24-Segment ED Z-Scores (EFW)[] Segment 14	85.29%	69.81%
LV 24-Segment ED Z-Scores (EFW)[] Segment 24	85.29%	84.91%
LV 24-Segment SI Z-Scores[] Segment 1	79.41%	75.47%
LV 24-Segment SI Z-Scores[] Segment 14	70.59%	62.26%
LV 24-Segment SI Z-Scores[] Segment 24	73.53%	71.7%
	Centiles (5–95) Agreement	
RV 24-Segment FS Centiles[%] Segment 1	58.82%	66.04%
RV 24-Segment FS Centiles[%] Segment 12	67.65%	58.49%
RV 24-Segment FS Centiles[%] Segment 24	82.35%	67.92%
LV 24-Segment FS Centiles[%] Segment 1	50%	50.94%
LV 24-Segment FS Centiles[%] Segment 12	58.82%	56.60%
LV 24-Segment FS Centiles[%] Segment 24	79.41%	64.15%

## Data Availability

The dataset supporting this study has been deposited in a public repository and is accessible at the following URL: https://doi.org/10.17605/OSF.IO/SFA7T (accessed on 26 March 2025).

## Supplementary Materials

The following supporting information can be downloaded at: https://doi.org/10.17605/OSF.IO/SFA7T (accessed on 26 March 2025), Supplementary file: fetalHQ.
